# Ultralow-Concentration Pt-Decorated Carbon Sphere Catalyst for Enhanced Hydrogen Evolution Reaction

**DOI:** 10.1021/acsomega.4c08324

**Published:** 2024-11-15

**Authors:** Naveen Kumar Reddy Bogireddy, Mohan Kumar Kesarla, Ana Laura Elías, Yu Lei, Rodolfo Cruz-Silva, Fu Zhang, He Liu, Mauricio Terrones, Vivechana Agarwal

**Affiliations:** † Instituto de Ciencias Físicas, Universidad Nacional Autónoma de México, Cuernavaca, Morelos C.P. 62210, Mexico; ‡ Department of Physics, Binghamton University, Binghamton, New York 13902, United States; § Department of Physics, Center for 2-Dimensional and Layered Materials, The Pennsylvania State University, University Park, Pennsylvania 16802, United States; ∥ Centro de Investigación en Química Aplicada, Blvd. Enrique Reyna #140, Saltillo, Coahuila C.P. 25294, Mexico; ⊥ Department of Materials Science and Engineering, The Pennsylvania State University, University Park, Pennsylvania 16802, United States; # Department of Material Science and Engineering, Centro de Investigación en Ingeniería y Ciencias Aplicadas, IICBA-Universidad Autónoma del Estado de Morelos, Av. Univ. 1001, Col. Chamilpa, Cuernavaca, Morelos 62209, Mexico

## Abstract

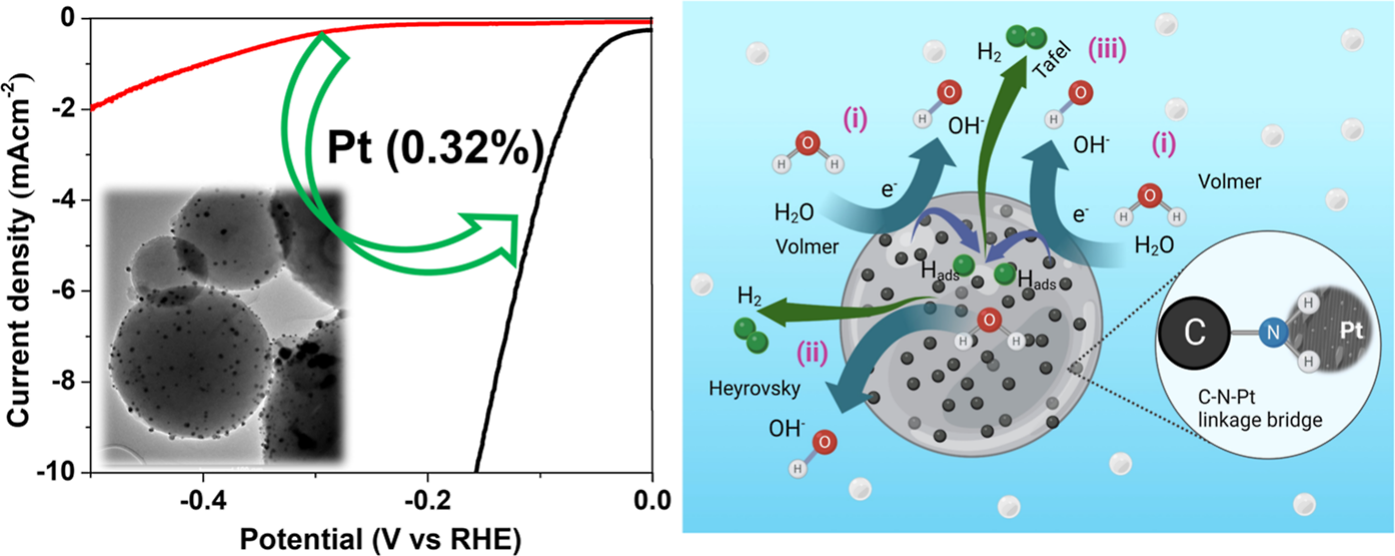

Ultralow decoration of platinum nanoparticles (Pt NPs) (0.32 wt %) onto carbon spheres (CS) has been developed for hybrid formation, using hydrothermal heat treatment, followed by chemical reduction of nanoparticles. The successful decoration of CSs bearing amine groups with platinum nanoparticles was confirmed directly by X-ray photoelectron spectroscopy. The uniform distribution and crystallinity of the Pt NPs in the hybrid structures were confirmed from X-ray diffraction spectroscopy and transmission electron microscopy. The hydrogen evolution reaction studied on the proposed Pt-CS hybrids reveals an onset potential of only −144 mV (vs RHE, reversible hydrogen electrode) with a current density of 10 mA/cm^2^ under an acidic 0.5 M H_2_SO_4_ medium, which exhibits a better performance as compared to a similar Pt-carbon hybrid catalyst reported in the literature. The Pt-CS hybrid stability assessments revealed a minimal overpotential loss (9 mV) from 144 to 153 mV after 200 cycles. Such hybrids have possible applications in environmental water purification and renewable energy production.

## 1 Introduction

Due to a lack of potential clean energy sources, there is a necessity to explore different promising renewable energy sources.^1−3^ In the persistent effort to replace fossil fuels (the world’s primary fuel source) with hydrogen, an extensive effort in developing, understanding, and improving the efficiency of electroactive hydrogen evolution reaction (HER) catalysts is being carried out by many groups of researchers.^4,5^ Addressing this challenge is critical for the global economy and will also help mitigate fossil fuels’ environmental and health impacts.^6−8^

The critical point for HER activity hinges on the effectual electron transfer on the catalyst’s surface and within the electrocatalyst itself. Even though this criterion is met by many ideal metal catalysts like Rh, Ru, Pd, Au, and Pt,^9−14^ due to their conducting nature, the lack of catalyst’s economic viability and abundancy limits their use as HER electrocatalysts. Many nonmetallic electrocatalysts, such as carbon, Mo, and transition metal-based electrocatalysts,^15−20^ were studied recently, and their performance was found to be far lower than the noble metal electrocatalysts. To overcome this situation, decreasing the amount of noble metal clusters on nonmetallic conducting support has been taken as one possible solution.^21−24^

Among the nonmetallic catalyst supports, carbon and its allotropes have been studied to support electrocatalytic HER for the last five decades.^25^ Carbon supports have various advantages such as (1) high abundancy, tunable structure, and durable fortitude in acidic/basic media; (2) resisting the noble metal aggregation, thereby increasing the active site availability to enhance the overall HER performance; and (3) their insoluble nature provides long life to the catalysts. Among the several carbon-based substrate materials, carbon spheres (CSs) gained more attention recently due to their high affinity toward platinum.^23^ It has been generally supposed that increasing the noble metal content on the substrates is a key to enhance hydrogen production.^26^ Zhang et al.^26^ presented platinum (0.53 wt %) confined into a mesoporous carbon matrix to produce 105 mV at 10 mA cm^–2^. From the density functional theory (DFT) calculations, the authors proposed that the presence of active sites is associated with the lattice-confined Pt centers and that the activated carbon (C)/nitrogen (N) atoms at the adjacency of the isolated Pt centers help improve the catalytic activity. Li et al.^27^ reported platinum (0.2 wt %) nanoclusters embedded in porous RF CSs to obtain ≈380 mV at 10 mA cm^–2^. The complicated mechanism for H_2_ formation occurs via a Tafel mechanism (H adsorption-recombination step) on Pt surfaces, and Pt shows the optimal level for H adsorption (step with a free energy of approximately zero) with respect to other metals which benefits H_2_ evolution. Yuan et al.^28^ reported an overpotential of 125 mV at 10 mA cm^–2^ using platinum (0.8 wt %) on the versatile Ti_3_C_2_T_*x*_ (MXene) and attributed the corresponding H_2_ evolution-enhancing mechanism to the charge transfer between O terminals and Pt. Similarly, Li et al.^29^ fabricated 0.5 wt % Pt NPs/MoO_2_ nanosheets synthesized by thermal reduction with a HER activity of 127 mV@10 mA/cm^2^. From the DFT calculations, the authors predict that the adsorption/desorption performance of H* is significantly improved on platinum and nearby molybdenum. Moreover, single-atom Pt doping in the form of a Pt–2H–MoS_2_ catalysts showed a distinctive HER enhancement.^30^ Xuan et al.^30^ reported an overpotential of 300 mV with Pt (1.2 at %)–2H–MoS_2_ at 10 mA/cm^2^, from the Tafel slope of 2H–MoS_2_, suggesting their homologous catalytic environment and mechanism.

In this work, we propose a novel electrocatalyst synthesized through a simple two-step synthesis of CSs, silanization (amine group functionalization), with APTES and decorated with noble metal (Ag, Au, and Pt) nanoparticles. For HER activity in 0.5 M H_2_SO_4_, the prepared Pt (0.32 wt %)-decorated CS hybrid catalyst reveals an enhanced electrocatalytic activity with 144 mV at 10 mA cm^–2^, which is better than the recently reported catalysts with a comparable platinum loading. From the obtained Tafel slope values, HER on Pt-CSs hybrids occurred through a Volmer–Heyrovsky mechanism.

## 2 Results and Discussion

**Figure 1:**
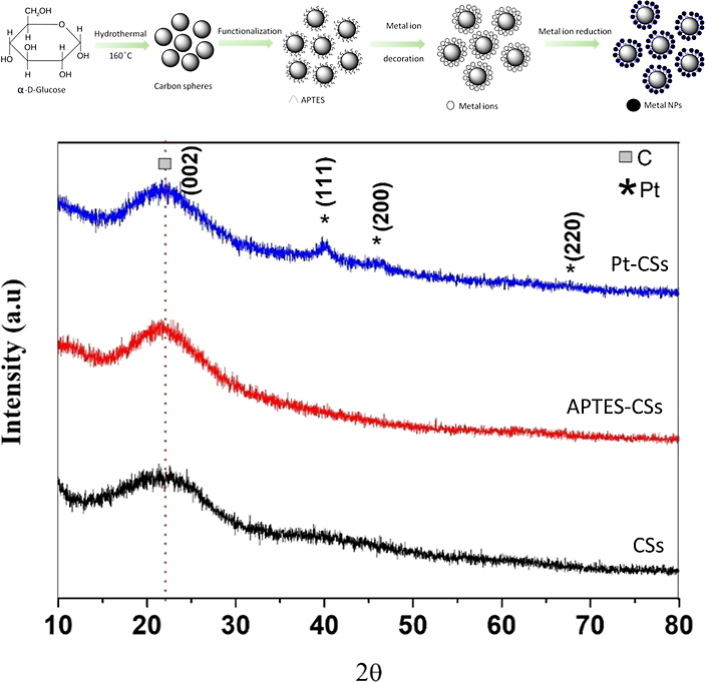
Step-by-step fabrication process representation of metal nanoparticle-decorated CSs (upper part) and their X-ray diffraction patterns of as-prepared carbon spheres (CSs; black), APTES with CSs (APTES-CSs; red), and platinum nanoparticles decorated onto APTES-functionalized CSs (Pt-CSs; blue).

The CSs were synthesized at 160 °C for 120 min by a hydrothermal method in the presence of α-d-glucose as the carbon precursor (Figure 1, upper panel). Subsequently, the salinization of fabricated CSs with 3-aminopropyl triethoxysilane (APTES) was conducted, which can attach metal ion species to form metal NPs by reduction with NaBH_4_. A brown-colored solution was obtained during the process, indicating NP formation. APTES enhanced the colloidal stability of the produced metal NP CSs since there was no possibility of precipitation in this state.

The XRD patterns of CSs, APTES-CSs, and Pt-CSs (Figure 1) show a broad peak at 2θ = 22.21°, corresponding to the (002) carbon peak of limited carbonization of the material, characteristic of a graphitic lattice. There is no significant change in the peak intensity at 22.21° across the tested samples, even after the functionalization of CSs with APTES. The incorporation of Pt nanoparticles on APTES-CSs results in the appearance of XRD peaks at 39.84, 46.34, and 67.52°, corresponding to the (111), (200), and (220) planes of Pt nanoparticles (JCPDS # 87-0647), respectively.^31^ The intensity of the peaks corresponding to Pt was low intensity, which indicates the possible low crystallinity of Pt throughout the CSs. Comparing them with the XRD patterns of Ag-CSs and Au-CSs [shown in the Supporting Information (Figure S1)], the more intense peaks of Au displayed in the Au-CSs XRD pattern could be due to a relatively better crystallinity level of Au in Au-CSs (with respect to that of Ag-CSs and Pt-CSs) or the formation of Au NP agglomerations onto the CSs.^32^

**Figure 2:**
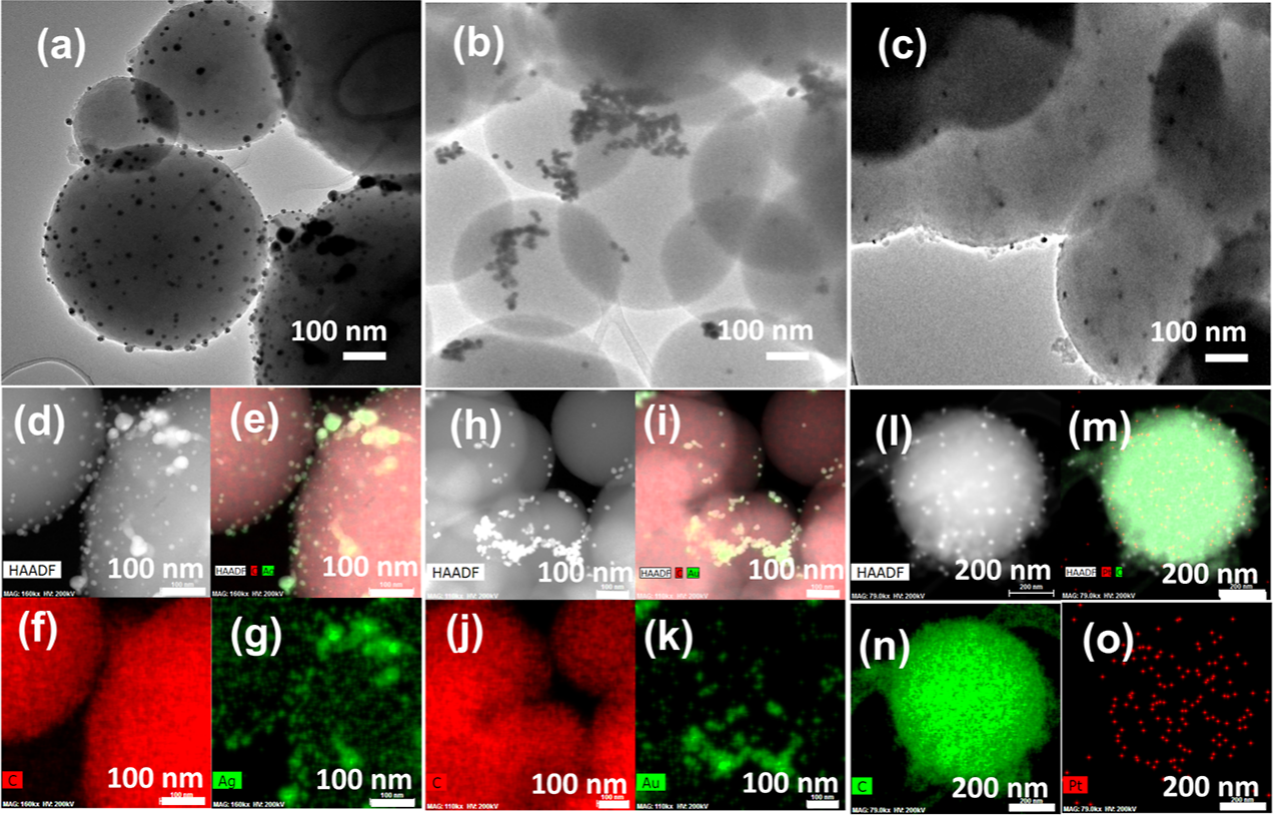
TEM along with EDS mapping images of silver nanoparticle-decorated CSs (Ag-CSs) (a,d–g), gold nanoparticle-decorated CSs (Au-CSs) (b,h–k), and platinum nanoparticle-decorated CSs (Pt-CSs) (c,l–o) respectively.

Transmission electron microscopy (TEM) was conducted in all of the pristine and metal-decorated CSs. The as-prepared CSs have a broad size distribution of 150 ± 100 nm (Figure S2 in the Supporting Information). The size distributions of the synthesized silver, gold, and platinum NPs (suspension) obtained from TEM images were found to be around 7 ± 5, 10 ± 3, and 8 ± 4, respectively, which confirms the relative uniformness in the size and shape distribution of the fabricated noble metal nanoparticles (Figure S3). The corresponding TEM images of individual metal nanoparticles decorated onto CSs are also presented in Figure 2a–c. The high-angle annular dark-field TEM image and the energy-dispersive X-ray spectroscopy (EDS) mapping of the as-prepared noble metal NP show the presence of carbon (C) from CS (in all hybrid structures), along with their respective decorated metal nanoparticles, i.e., silver (Ag), gold (Au), and platinum (Pt) of Ag-CSs, Au-CSs, and Pt-CSs, respectively (Figure 2d–o). Similar morphological characteristics were confirmed through field emission scanning electron microscopy results (Figure S3). The Brunauer–Emmett–Teller (BET) surface of the synthesized CSs was found to be 20.15 m^2^/g (Figure S4).

**Figure 3:**
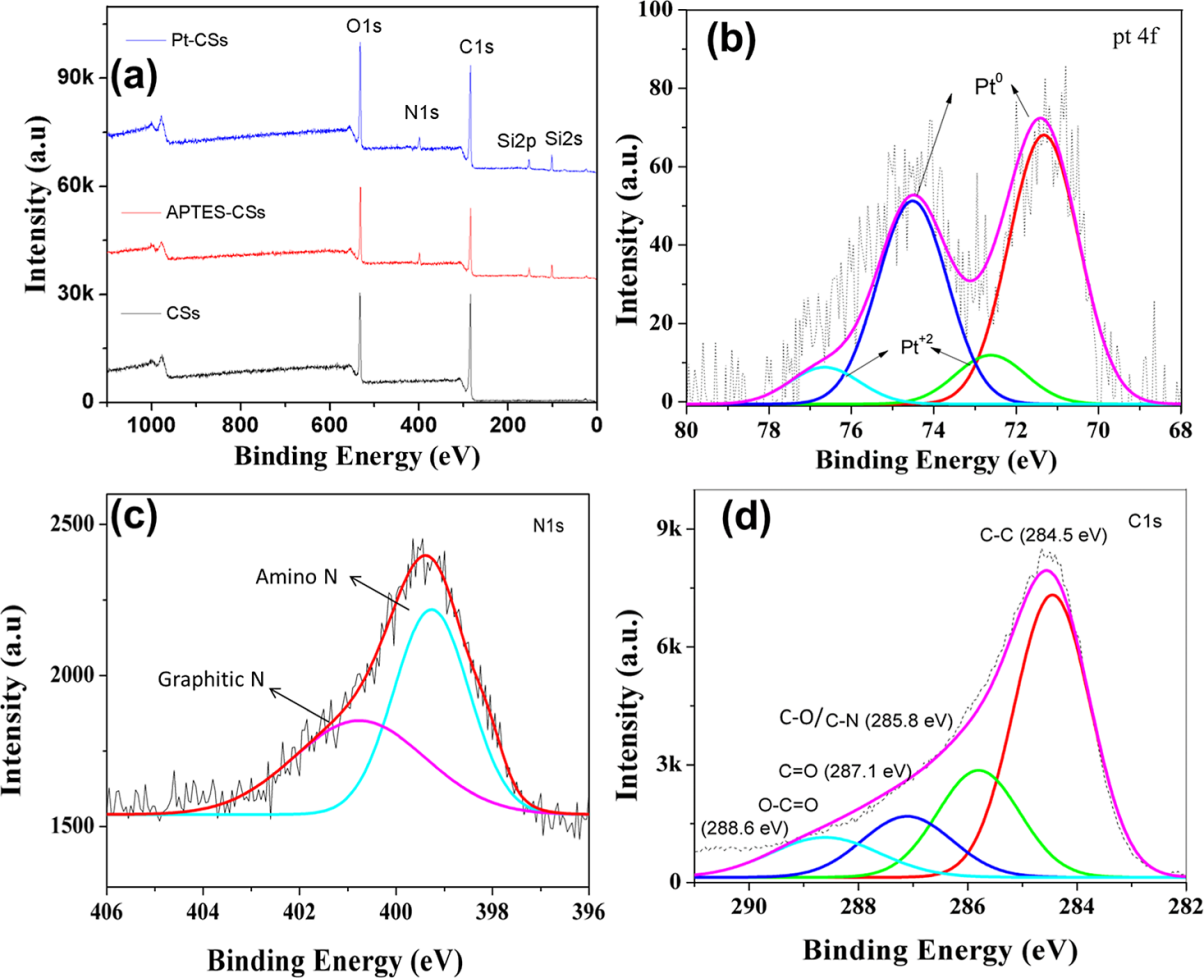
(a) XPS survey scan (CSs, black; APTES-CSs, red; and Pt-CSs, blue) and (b–d) deconvoluted XPS spectra corresponding to sample Pt-CSs (Pt 4f, N 1s, and C 1s).

X-ray photoelectron spectroscopy (XPS) survey scans of CSs, APTES-CSs, and Pt-CSs are shown in Figure 3a, confirming the presence of Si 2p signals (around 102 eV) in APTES-CSs and Pt 4f and Si 2p signals in Pt-CSs, which validates the successful interaction of platinum with APTES-CSs due to the presence of plenty of –OH residual groups in CSs that can react with silanol groups from the silane. The high-resolution XPS spectra in the Pt 4f regions showed two distinct overlapping peaks, corresponding to Pt 4f_7/2_ and Pt 4f_5/2_, respectively (Figure 3b). Further, the signal was deconvoluted into four peaks centered at 71.3, 72.6, 74.5, and 76.6 eV, in which 71.3 and 74.5 eV can be ascribed to metallic Pt^0^, while 72.9 and 76.1 eV correspond to a metal oxide Pt^2+^ state, respectively. It is clear that Pt^0^’s metallic state predominates over the Pt oxide form in the synthesized catalyst. It is noteworthy that the Pt 4f line scan showed a shift toward higher binding energy when compared to standard/commercial Pt–C (70.3 eV for 4f_7/2_ and 74.0 eV for 4f_5/2_),^33^ which is indicative of the interactions between the amino groups of APTES-functionalized CSs and Pt nanoparticles. This interlinkage is critical to preserve the enduring catalytic abilities by keeping the Pt NPs well dispersed on the supporting CS substrate.^34^Figure 3c shows high-resolution N 1s spectra of Pt-CSs in which two peaks at 399.25 and 400.79 eV suggest the presence of pyridinic and graphitic nitrogen in Pt-CSs.^34^ Deconvoluted high-resolution C 1s spectra of the Pt-CS catalyst are shown in Figure 3d. The deconvoluted peaks at 284.5, 285.4, 287.1, and 288.6 eV suggest the presence of C–C, C–N, C=O, and −O–C=O linkages, respectively. In general, the XPS analysis supports the successful decoration of Pt NPs on APTES-functionalized CSs. The XPS peaks estimated at % of C, O, N, Si, and Pt in Pt-CSs are around 66.6, 22.3, 3.6, 7.4, and 0.1 at %, respectively.

Further, the functional groups present in the hybrid systems were analyzed using Fourier transform infrared (FT-IR) spectroscopy. The CSs, APTES-CSs, and metal nanoparticle (Pt, Ag, and Au)-decorated CS FT-IR spectra are shown in Figure S5a. Various distinctive absorption bands were observed in all of the samples. In CSs (Figure S5a; black curve), the bands at 3402 and 1030 cm^–1^ refer to the presence of hydroxy (O–H) and C–OH stretching, respectively. The C–H stretching can be seen clearly at 2941 cm^–1^. The peak around 1700 cm^–1^ is due to the presence of C=O stretching from the –COOH functionalization. The peaks at around 1287 and 1610 cm^–1^ are due to C–C and C=C stretching, respectively. The peaks around 750–920 cm^–1^ correspond to aromatic C–H bending. The observations show that there are plenty of functionalities in the synthesized CSs.

After the CSs are functionalized with APTES (Figure S5a; rose curve), the C=O peak previously observed around 1700 cm^–1^ in CSs completely disappears. The C–N stretching vibrations can be observed around 1390 cm^–1^ from APTES-CSs, which indicates the interaction of amino groups with the carboxylic groups of CSs. The peak around 1040 cm^–1^ is attributed to the presence of Si–O–C originating from salinization on the surface of CSs. After immobilization of metal nanoparticles (Ag, Au, and Pt) (Figure S5a; blue, green, and red curves), spectra display similarities to the APTES-CS spectrum. Particularly, a peak at 910 cm^–1^ appears once again in these hybrids, indicating the successful interaction of metal nanoparticles with APTES-functionalized surfaces and exposing back the aromatic C–H bending from the carbon surface. This observation shows the successful functionalization of CSs with APTES and simultaneous decoration of metal nanoparticles on the surface of the CSs.

The amount of platinum on the CSs was determined by thermogravimetric analysis (TGA), as shown in Figure S5b. The APTES-CSs had a high content of APTES (approximately 23% by wt) from TGA; according to our synthesis parameters, they have an APTES content of 23% by wt % (since APTES after hydrolysis has an *M*_w_ value of 86 g/mol and results in 60 g/mol of SiO_2_). Both the APTES-CSs and the Pt-CSs have a higher oxygen content when compared to CSs and lose 40 wt % at just 400 °C (i.e., 50% for the pure CSs without APTES). In the case of CSs, a complete weight loss was observed after 500 °C, which is expected in CSs alone. When functionalized with APTES, there is no complete weight loss due to the formation of SiO_2_ from APTES that remained after 625 °C. The weight % of platinum was estimated to be 0.32% from the difference in remaining weights after 720 °C from the APTES-CSs and Pt-CSs. The Pt concentrations obtained from the XPS and TGA coincide with the solution-mode inductively coupled plasma mass spectrometry results [Pt (0.33 wt %)] onto CSs.

### 2.1 HER Performance

**Figure 4:**
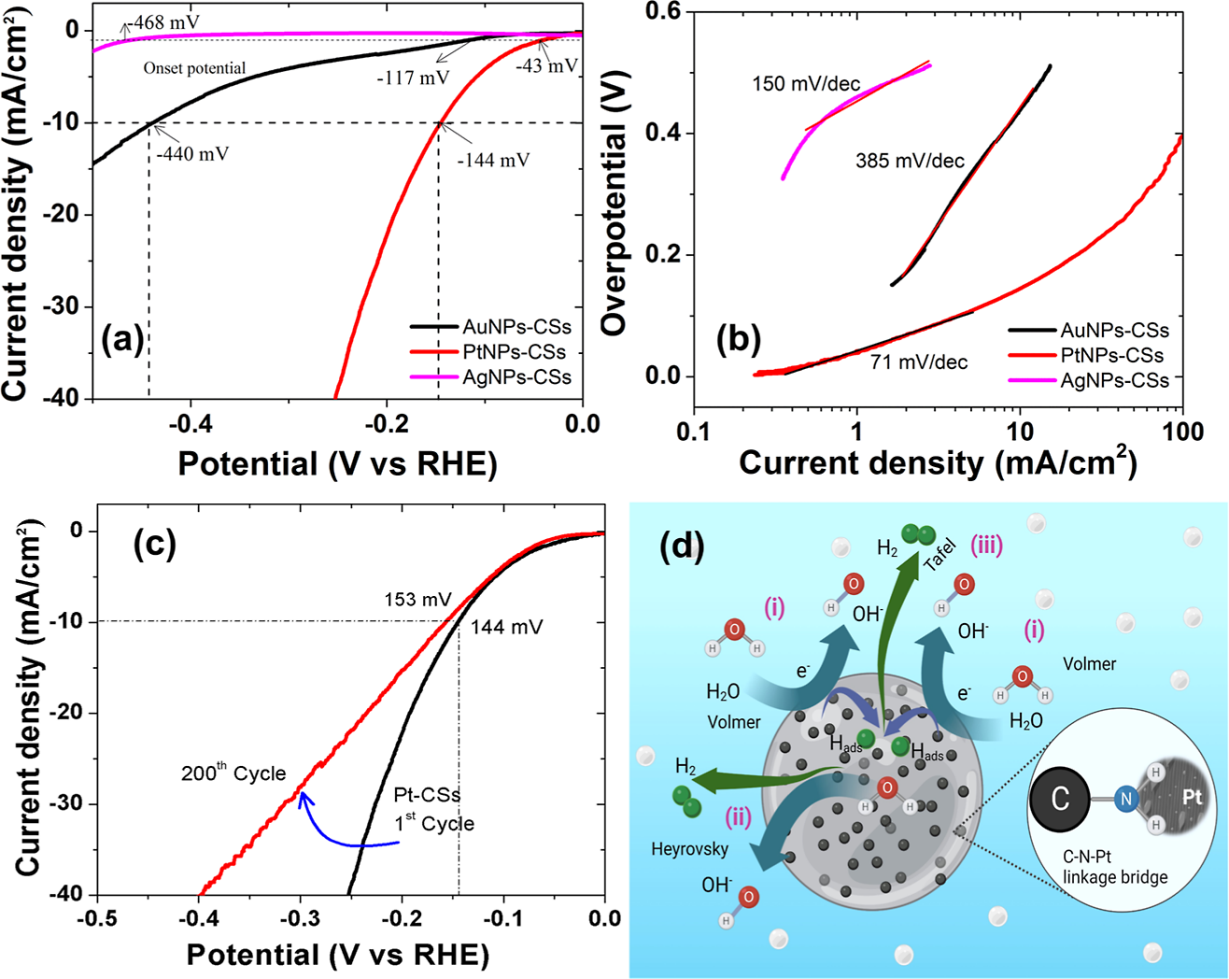
Optimized electrocatalytic hydrogen evolution performance: (a) LSV polarization curves; (b) Tafel slopes of Ag-CSs, Au-CSs, and Pt-CSs in a 0.5 M H_2_SO_4_ aqueous solution; (c) the LSV curves of Pt-CSs before and after 200 cycles; and (d) their possible mechanism representation.

The hydrogen evolution performance of the individual noble metal nanoparticle (AgNPs, AuNPs, and PtNPs)-decorated CSs was tested in a 0.5 M H_2_SO_4_ solution (Figures 4, S6, and S7). From Figure 4a, Pt-CSs (144 mV vs RHE) showed lower overpotentials when compared to Au-CSs (440 mV vs RHE) and Ag-CSs (468 mV vs RHE) at 1 mA cm^–2^, respectively. Current density is calculated using the formula *J* = *I*/*A*, where *J* is the current density, *I* is the current in mA, and *A* is the area of the electrode in cm^2^. The nominal geometric surface area is 0.16 cm^2^. The Pt-CSs exhibits smaller overpotentials than those of other catalysts reported in the literature, such as MoS_2_–Pt sheets (199 mV),^35^ Ru–MoS_2_ (≈270 mV),^36^ 1% Pd–C (≈200 mV),^37^ and Au–MoS_2_ (≈250 mV),^38^ respectively. This superior catalytic activity is probably due to the strong interaction between the amine groups of APTES-functionalized CSs and Pt nanoparticles, providing a good surface area and more reactive species to participate in the HER. In general, the rich coordination site in the nitrogen-functionalized carbon matrix favors the formation of well-dispersed small Pt nanoparticles on the CSs. The coordination of Pt with the adjacent C/N can increase the electronic metal support interaction (EMSI), as mentioned by Campbell.^39^ Nitrogen functionalization (APTES) can suppress the aggregation of Pt nanoparticles to increase the catalytic efficiency, and the undoped carbon fails to control the aggregation. The strong EMSI can be expected from C–N–Pt linkage bridges, which can predominantly tailor the Pt 5d states by facilitating the proton reduction/H–H coupling^40^ with overall enhancement in HER. A strong EMSI is also in good agreement with the XPS line scans.

Moreover, we performed polarization curve tests on the initial and 200th cycles (Figure 4c) with a sweeping potential range (0 to −0.5 V) and the highest current density (−40 mA/cm^2^). The Pt-CS catalyst stability assessments, conducted over a significant number of cycles, revealed a minimal overpotential loss (9 mV) from 144 to 153 mV after 200 cycles, confirming the catalyst’s stability.

It has been a common practice to use Tafel analysis to study and understand the HER mechanism.^38^ Generally, in basic media, three different possible types of reactions have been suggested for HER: (i) the Volmer reaction (H_3_O^+^ + e^–^ → H* + H_2_O), which represents the adsorption of hydrogen from hydronium ions, followed by (ii) a Heyrovsky reaction, an electrochemical desorption step (H* + H_3_O^+^ + e^–^ → H_2_ + H_2_O), or by (iii) the Tafel reaction, where the recombination takes place (H* + H* → H_2_).

The Tafel equation is given as follows
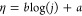
where η is the overpotential, *j* is the current density, and *a* and *b* are constants.

Based on the above equation, the Tafel slopes have been theoretically estimated as ∼30, ∼40, and ∼120 mV/dec for the Tafel reaction, Heyrovsky reaction, and Volmer reaction, respectively.^38^

For the present study, the Tafel slopes were plotted from the polarization curves presented in Figure 4b. The Pt-CSs exhibit a lower Tafel slope of 71 mV dec^–1^ when compared to those of Ag-CSs (385 mV dec^–1^) and Au-CSs (150 mV dec^–1^). In the present case, the Volmer–Heyrovsky catalytic reaction mechanism is observed and can be compared with the already established theoretical calculations (Figure 4d).^41−43^ Moreover, Pt-CSs exhibit a smaller Tafel slope than that of 1% Pd–C and Au–MoS_2_ corresponding to 100, 135, and 78 mV dec^–1^,^37,38^ respectively, which means a lower over potential is needed to achieve efficient hydrogen production.

## 3 Conclusions

In conclusion, we present the successful decoration of ultralow (0.32 wt %) platinum nanoparticles onto CSs (125 times lower than commercial 40 wt % Pt/C) for electrocatalytic applications. The measured superior electrocatalytic performance toward HER through the proposed hybrids has been attributed to several major factors. An important one is that the uniform distribution of small-sized PtNPs (8 ± 4 nm) onto CSs allows enhanced availability of the reactive species. Additionally, silane functionalities play a vital role in binding metal nanoparticles onto CSs. Moreover, the strong EMSI can be expected from C–N–Pt linkage bridges, which can predominantly tailor the Pt 5d states by facilitating superior HER activity. The present study exhibits that the ultralow concentration of Pt-decorated CSs is an effective strategy for the design of novel catalysts with enhanced HER performances, with minute amounts of earth scarce Pt and environmentally friendly carbon. These results constitute a promising path toward the development of hydrogen energy/battery prototypes.

## 4 Materials and Methods

In this work, α-d-glucose, (3-aminopropyl) triethoxysilane (APTES), absolute ethanol (99%), hexachloroplatinic acid (H_2_PtCl_6_, 8 wt % in H_2_O), chloroauric acid (HAuCl_4_·6H_2_O, 99.9% trace metal basis), silver nitrate (AgNO_3_, 99.9% trace metal basis), sodium citrate (Na_3_C_6_H_5_O_7_·2H_2_O, ACS reagent, ≥99.0%), and sodium borohydride (NaBH_4_, 99% reagent plus) were purchased from Sigma-Aldrich Co., Ltd. and used without further treatment.

### 4.1 Synthesis of CSs

The spherical carbon supports were synthesized hydrothermally by using α-d-glucose as a carbon source. At room temperature, 3.7 g of α-d-glucose dispersed in 40 mL (total volume of the beaker, 100 mL) with H_2_O to form a transparent solution was kept under vigorous magnetic agitation for 0.5 h. The mixture was heated hydrothermally in an autoclave at 160 °C for 120 min to obtain the desired CSs. The obtained CSs were centrifuged more than eight times with a combination of ethanol and water to remove unreacted precursors present with CSs, and finally, a solid black powder was obtained.

### 4.2 Synthesis of Amine-Functionalized CSs

To obtain amino group-functionalized CSs,^10,32^ 10 mL of a 1:9 volume ratio of (3-aminopropyl) triethoxysilane and ethanol (99%) was added to 10 mg of CSs. The CSs and APTES mixture was stirred under ambient conditions overnight. The subsequent amine group-functionalized CSs were separated by centrifugation at 6000 rpm for 30 min and washed several times by alternating H_2_O and ethanol. Then, the obtained black powder was redispersed in 5 mL of distilled water.

### 4.3 Synthesis of Noble Metal Nanoparticle-Decorated CS Hybrid Structures

About 2 mL of noble metal ion solution dispersion (1 mM) was added to 10 mg of amine-functionalized CSs under magnetic stirring overnight under ambient conditions. Subsequently, the metal nanoparticles decorated with CSs were separated by centrifugation at 6000 rpm to remove excess and remnant metal nanoparticles. The hybrid structures had the visual appearance of a fine black powder and were stored under ambient conditions for further use.

### 4.4 Electrochemical Hydrogen Evolution Measurement

H_2_ production measurements were carried out using a three-electrode electrochemical setup with Ag/AgCl (3 M NaCl) as a reference electrode, graphite rod as a counter electrode, and noble metal nanoparticle-decorated CSs as catalysts (Ag-CSs, Au-CSs, and Pt-CSs) as well as the working electrode, using a VersaSTAT4 potentiostat.^44^ The reference electrode was standardized for RHE in the presence of a high-purity H_2_ saturated 0.5 M H_2_SO_4_ electrolyte. A 5:1 ratio of hybrid catalyst, mixed with carbon powder and suspended in 1:2:8 volume ratios of Nafion/distilled water/isopropanol mixture, was sonicated for 1 h to form a slurry. Later, it was deposited onto a 3 mm working electrode.

## Supporting Information Available

Characterization information; XRD spectra of Ag-CSs and Au-CSs; scanning electron micrographs of CSs, silver nanoparticle-decorated CSs, gold nanoparticle-decorated CSs and platinum nanoparticle-decorated CSs; size distribution histogram for AgNPs, AuNPs, and PtNPs measured from transmission electron micrographs; the BET surface properties of the synthesized CSs; FT-IR analysis of CSs, amine functionalized CSs, Ag-CSs, Au-CSs; green and Pt-CSs nanoparticles, respectively; thermogravimetric analysis of CSs, APTES-CSs, and Pt-CSs; HER data was processed using MATLAB program; electrocatalytic LSV polarization curves for glassy carbon electrode and carbon powder; hydrogen evolution performance optimization using metal nanoparticles on CSs: polarization curves of AgNPs-CSs, AuNPs-CSs and PtNPs-CSs in a 0.5 M H_2_SO_4_ electrolyte and their Tafel plots derived from the corresponding polarization curves presented in the Supporting Information (PDF)
