# Supplementary material for: Ultralow-Concentration Pt-Decorated Carbon Sphere Catalyst for Enhanced Hydrogen Evolution Reaction
Source: ACS Omega. 2024 Nov 15;9(47):47254–60. doi: 10.1021/acsomega.4c08324 (PMC11603201; doi:10.1021/acsomega.4c08324)
Supplement: Supplementary file 1 — ao4c08324_si_001.pdf [file ao4c08324_si_001.pdf]

Ultra-low Concentration Pt Decorated Carbon Sphere Catalyst for Enhanced Hydrogen Evolution Reaction

*Naveen Kumar Reddy Bogireddy, *[a] Mohan Kumar Kesarla, [a] Ana Laura Elías, [b] Yu Lei, [c] Rodolfo Cruz-Silva, [d] Fu Zhang, [e] He Liu, [e] Mauricio Terrones, *[c, e] and Vivechana Agarwal *[f]*

[a]Instituto de Ciencias Físicas, Universidad Nacional Autónoma de México, C.P 62210, Cuernavaca, Morelos, México.

[b] Department of Physics, Binghamton University, Binghamton, NY 13902, USA.

[c] Department of Physics, Center for 2-Dimensional and Layered Materials, The Pennsylvania State University, University Park, PA 16802, USA.

[d] Centro de Investigación en Química Aplicada, Blvd. Enrique Reyna #140, C.P. 25294, Saltillo, Coahuila, Mexico.

[e] Department of Materials Science and Engineering, The Pennsylvania State University, University Park, Pennsylvania 16802, United States.

[f]Department of Material Science and Engineering, Centro de Investigación en Ingeniería y Ciencias Aplicadas, IICBA- Universidad Autónoma del Estado de Morelos, Av. Univ. 1001, Col. Chamilpa, Cuernavaca, Morelos, 62209, México.

Characterization

X-ray diffraction (XRD) analysis of the hybrid structures was carried out in a Bruker D8 Advance eco diffractometer, using Cu Kα (λ =1.5406 Å) radiation. The size, morphology, and composition of the metallic nanoparticles were studied using high-resolution transmission electron microscopy (TEM) images, acquired using JEOL JEM 2100 equipped with an energy dispersive spectroscopy (EDS) system and the fabricated catalysts also studied in a Schottky field emission scanning electron microscope (FESEM, Hitachi SU5000). The ESCA Ulvac-PHI 1600 photoelectron spectrometer was used to carry out surface sensitive, quantitative XPS analyses, with Al Kα radiation photon energy (1486.6 ± 0.2 eV) and the full width half maximum (FWHM) of all deconvoluted spectra maintained at 1.52±0.03 eV (using origin 2021b). Some surface characteristics of the synthesized nanoparticles were analyzed through Varian 660-IR FT-IR spectrophotometer. The thermogravimetric (TG) analysis was performed on Rigaku Therm plus TG8120 at a heating rate of 10 ^{o}C min^{-1} in air. The specific surface area of the carbon spheres was determined through Brunauer–Emmett–Teller (BET) method using a five-point adsorption isotherm in the relative pressure range of P/P0 = 0.05/0.3 at 77.35 K. The ICP-MS analysis was performed by the plasma source mass spectrometry method with model iCAP Qc-Thermo Scientific instrument, which was optimized before the analysis of the samples, with a certified aqueous solution of the high purity standards brand (SM-1595-143). The calibration curve was made with 15 points (0 to 250 µg/L) from multi elemental stock solution of Au, Ir, Os, Pd, Pt, Rh, Ru, and Ag (ICP-MS-68A-Solution C) of 10 mg/L.

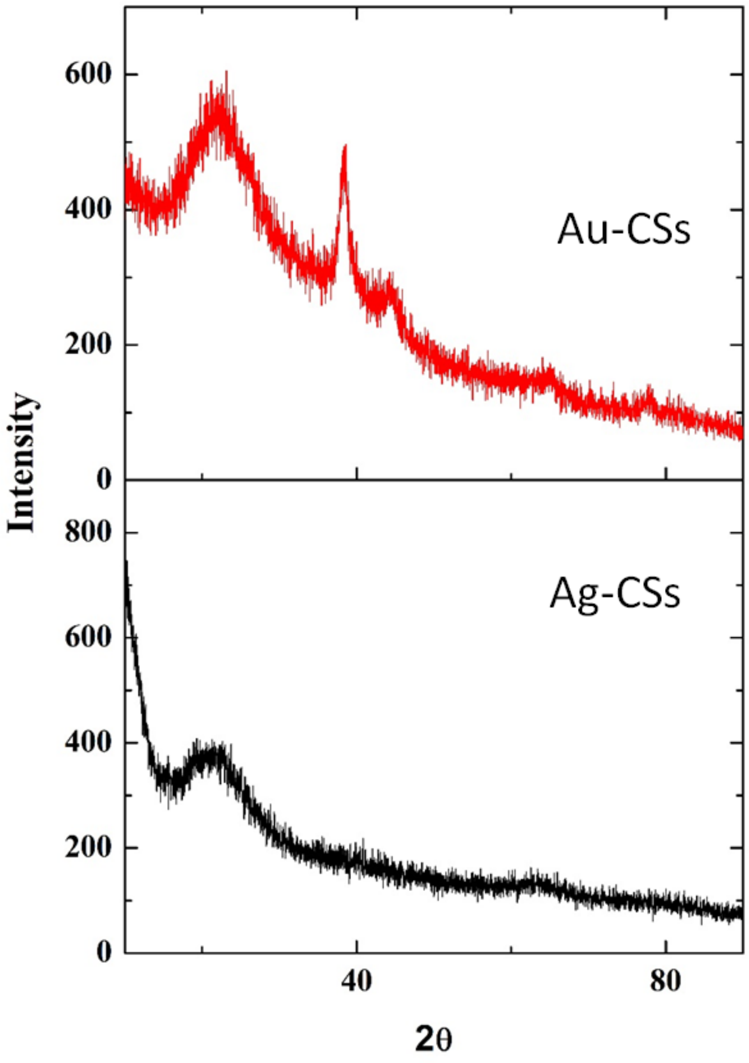

Figure S1. XRD spectra of Ag-CSs (black) and Au-CSs (red)

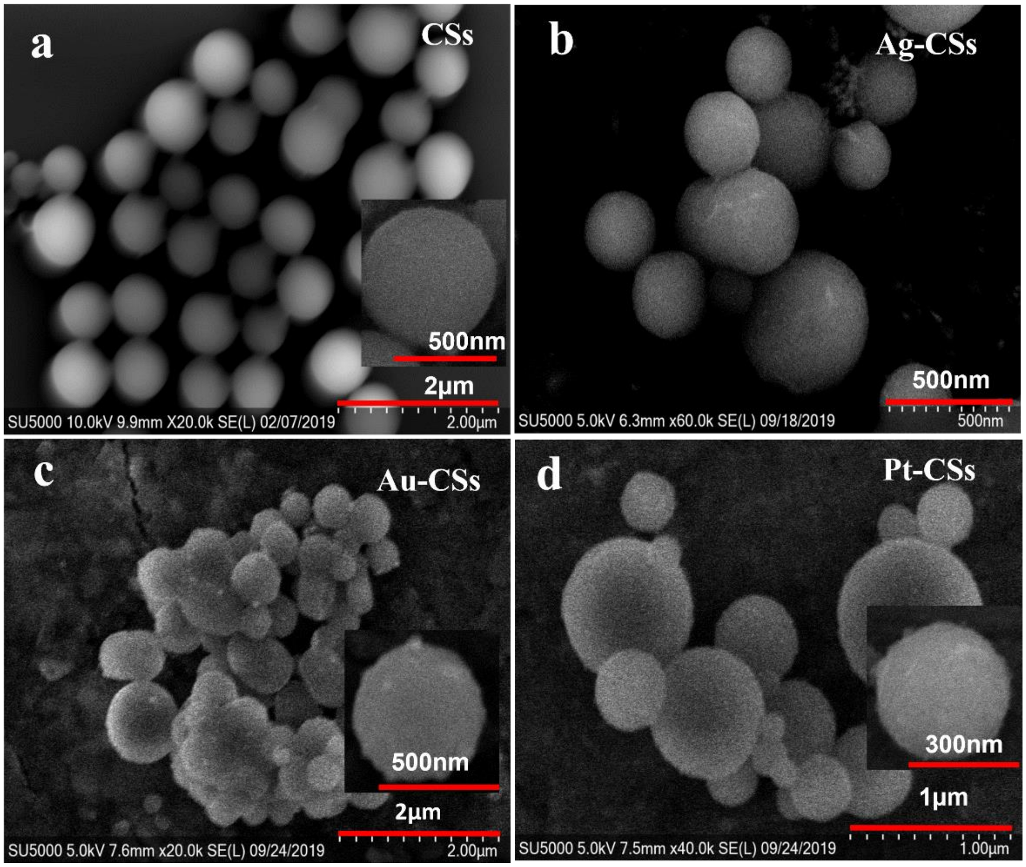

Figure S2. Scanning electron micrographs of (a) carbon spheres (CSs), (b) silver nanoparticles (Ag) decorated CSs (Ag-CSs), (c) gold nanoparticles (Au) decorated CSs (Au-CSs) and (d) platinum nanoparticles (Pt) decorated CSs (Pt-CSs) (inset show the corresponding high resolution SEM micrographs).

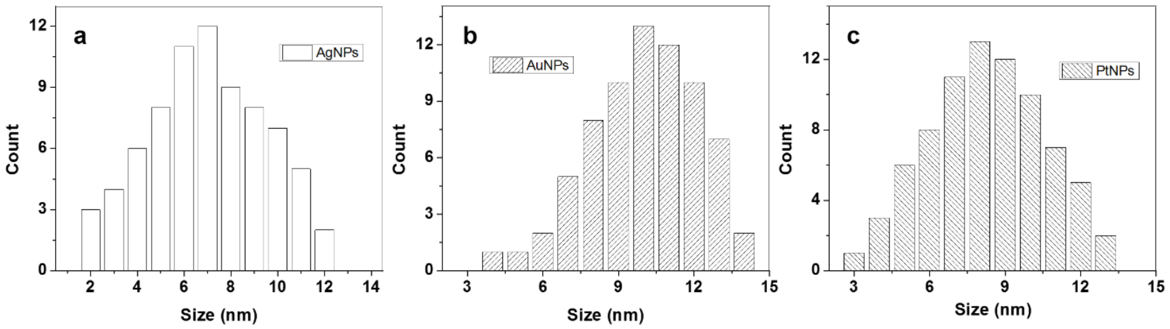

Figure S3. Size distribution histogram for AgNPs (a), AuNPs (b), and PtNPs (c) measured from transmission electron micrographs.

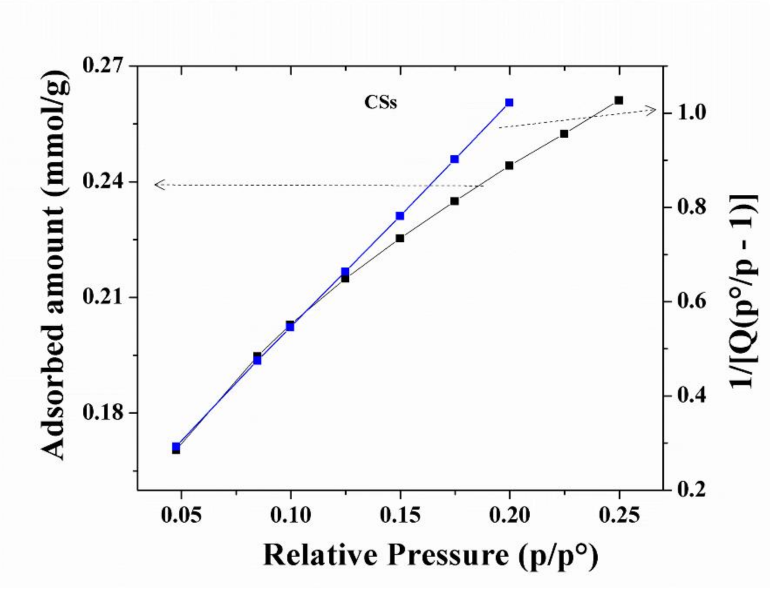

Carbon spheres	
BET Surface Area (m²/g)	20.15 ± 0.05
Slope (g/mol)	4.77 ± 0.01
Y-Intercept (g/mol)	0.06 ± 0.001
C	70.94
Q_m (mmol/g)	0.20
Correlation Coefficient	0.999
Molecular Cross-Sectional Area (nm²)	0.162

Figure S4. The Brunauer–Emmett–Teller (BET) surface properties of the synthesized carbon spheres

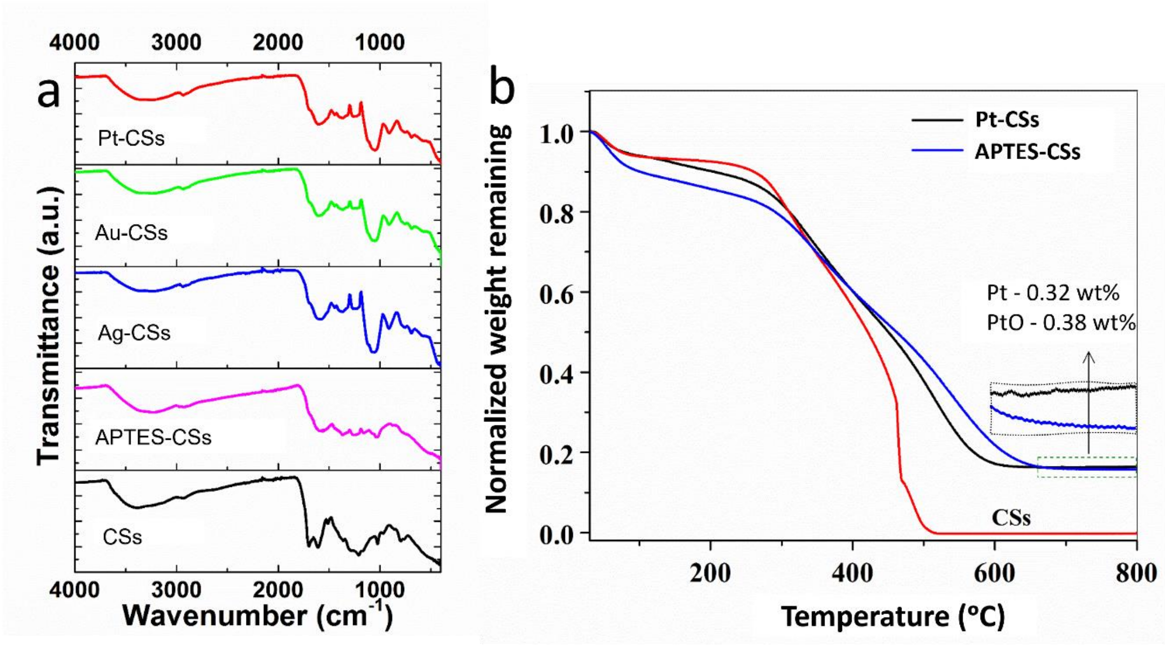

Figure S5. (a) FT-IR analysis of carbon spheres (CSs; black), amine functionalized carbon spheres (APTES-CSs; rose), silver (Ag-CSs; blue), gold (Au-CSs; green) and platinum (Pt - CSs; red) nanoparticles decorated carbon spheres, respectively. (b) Thermo-gravimetric analysis of CSs (red), APTES-CSs (blue), and Pt-CSs (black).

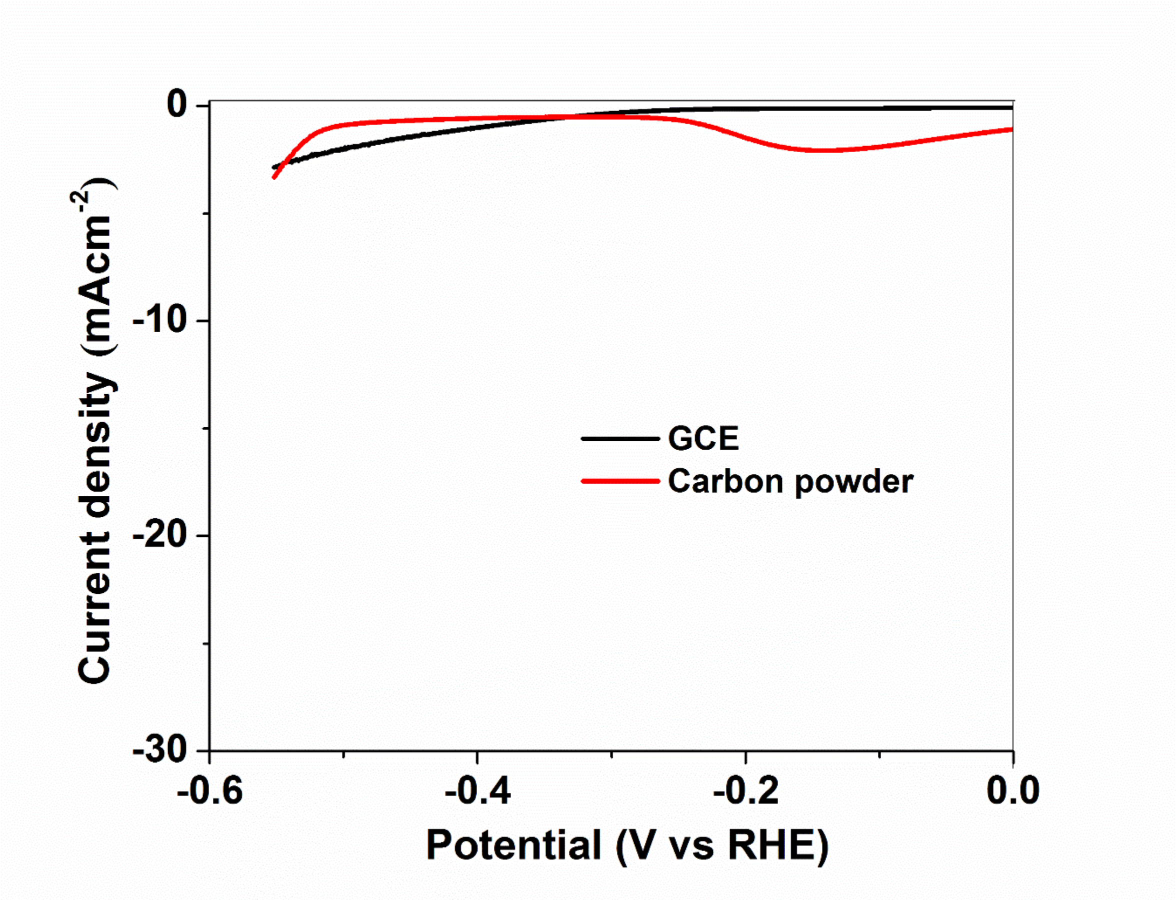

Figure S6. Electrocatalytic Linear sweep voltammetry (LSV) polarization curves for glassy carbon electrode (GCE, black) and carbon powder (red)

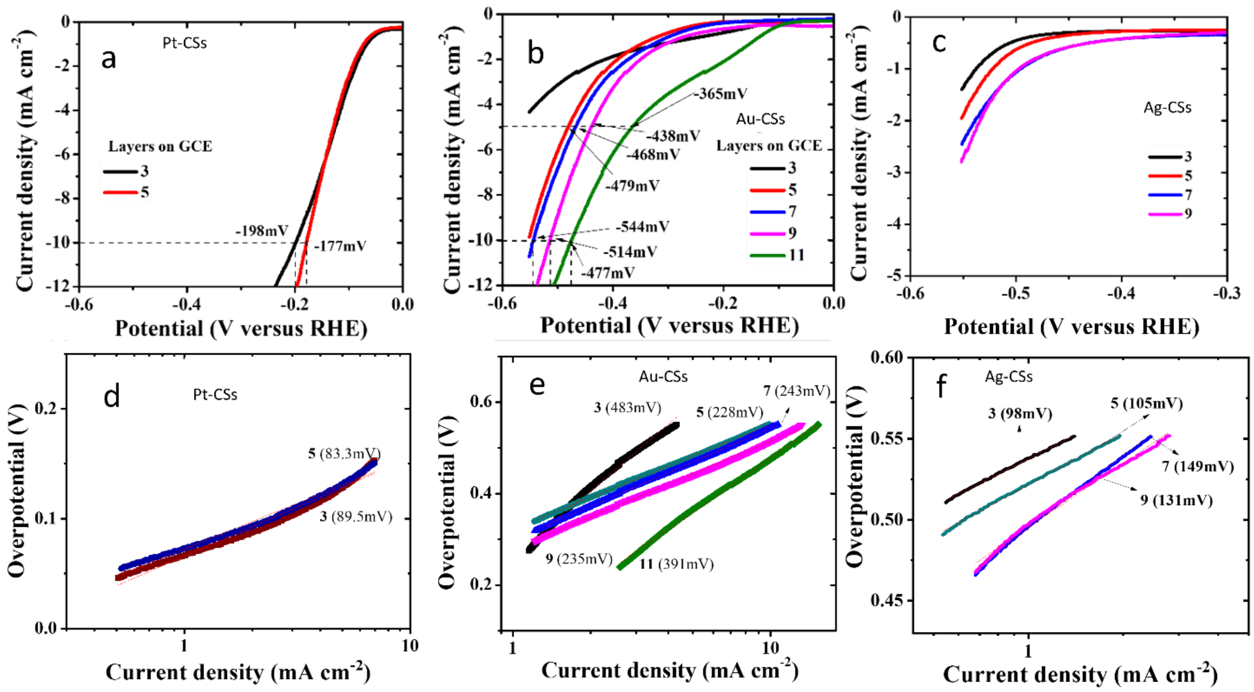

Figure S7. Hydrogen Evolution performance optimization using metal nanoparticles on CSs: Polarization curves of PtNPs-CSs (a and d), AuNPs-CSs (b and e) and AgNPs-CSs (c and f), in a 0.5M H_2SO_4 electrolyte. And their Tafel plots derived from the corresponding polarization curves.
